# Integrating Leg‐Press Strength and Nutritional Status Improves 16‐Year Mortality Risk Stratification in General Older Adults

**DOI:** 10.1002/jcsm.70375

**Published:** 2026-09-16

**Authors:** Yilin Du, Tatsuma Okazaki, Xiang Li, Keisuke Obata, Naoki Suzuki, Takahiro Miura, Midori Miyagi, Ryoichi Nagatomi, Mana Kogure, Naoki Nakaya, Atsushi Hozawa, Satoru Ebihara

**Affiliations:** ^1^ Department of Rehabilitation Medicine Tohoku University Graduate School of Medicine Sendai Japan; ^2^ Designing Future Health Initiative, Promotion Office of Strategic Innovation Tohoku University Sendai Japan; ^3^ Department of Preventive Medicine and Epidemiology Tohoku Medical Megabank Organization Sendai Japan; ^4^ Division of Epidemiology, Graduate School of Medicine Tohoku University Sendai Japan

**Keywords:** all‐cause death, leg‐press strength, MS‐GNRI, muscle strength, nutrition, sarcopenia

## Abstract

**Background:**

Malnutrition and muscle weakness are critical prognostic factors for mortality in older adults. While indices like the Geriatric Nutritional Risk Index (GNRI) are well‐established, the long‐term prognostic value of integrating nutrition with muscle strength (MS) remains insufficiently established in community‐dwelling populations. Currently, very few studies have evaluated the effects on mortality of directly measured leg‐press strength, which includes most of the major leg muscles in both lower extremities and involves a substantially larger muscle volume than handgrip or quadriceps muscle strength. This study aimed to clarify whether directly measured leg‐press strength predicts mortality and whether integration with GNRI (MS‐GNRI) enhances mortality risk stratification.

**Methods:**

We analysed data from 1065 Japanese community‐dwelling individuals aged ≥ 70 years from the Tsurugaya Project, followed for 16 years. Participants were categorized into two groups based on optimal cutoff values for directly measured leg‐press strength adjusted for body size (high and low) or by GNRI (poor and good), respectively. Finally, these baseline classifications were integrated to stratify participants into the following four MS‐GNRI groups: (1) high‐strength/good‐GNRI, (2) high‐strength/poor‐GNRI, (3) low‐strength/good‐GNRI and (4) low‐strength/poor‐GNRI. Hazard ratios (HRs) for all‐cause mortality were calculated using Cox proportional hazards models.

**Results:**

During the follow‐up, 415 deaths occurred. Low leg‐press strength and poor GNRI were independently associated with higher mortality than high strength and good GNRI, respectively; adjusted HRs were 1.81 (95% CI: 1.45–2.25) and 1.59 (1.30–1.95), respectively (both *p* < 0.01). The MS‐GNRI revealed a survival gradient: Using the high‐strength/good‐GNRI group as the reference, adjusted HRs (95% CI) were 1.55 (1.18–2.03) for the high‐strength/poor‐GNRI group, 1.69 (1.29–2.23) for the low‐strength/good‐GNRI group and 2.47 (1.78–3.13) for the low‐strength/poor‐GNRI group (*p* for trend < 0.001). Notably, the high‐strength/poor‐GNRI group and the low‐strength/good‐GNRI group showed comparable survival probabilities. MS‐GNRI significantly improved predictive accuracy and risk reclassification over either indicator alone (Net Reclassification Improvement: 0.62 over strength; 0.41 over GNRI).

**Conclusions:**

Directly measured leg‐press strength is a potent predictor of all‐cause mortality. Integrating muscle strength with nutrition via MS‐GNRI enhanced mortality risk stratification, suggesting that preserved muscle strength may substantially contribute to longevity alongside nutritional maintenance.

## 1 Introduction

Malnutrition in older adults is a key prognostic factor for total mortality [1, 2]. In older adults, the Geriatric Nutritional Risk Index (GNRI) is a well‐established clinical indicator for malnutrition. Determined by integrating serum albumin levels with the ratio of actual to ideal body weight (derived from height), the GNRI was originally developed to predict malnutrition‐related complications [3].

Concurrently, muscle weakness is associated with an elevated risk of total mortality [4]. Consequently, the coexistence of malnutrition and weakened muscle strength represents a critical prognostic factor for mortality. However, previous studies investigating the combined impact of these two factors have primarily focused on patients, such as those hospitalized or with cancer [1, 5]. Currently, the long‐term prognostic value of integrating nutrition and muscle strength for predicting all‐cause mortality in community‐dwelling older adults remains insufficiently established. While two prior studies combined both, the follow‐up period was relatively short at 4 or 5 years [6, 7]. Two studies from Thailand and Japan analysed total mortality by combining body mass index (BMI) with quadriceps or handgrip strength [8, 9]. However, the application of BMI as a nutritional indicator remains a subject of ongoing debate [10].

Handgrip, quadriceps and respiratory muscle strength serve as reliable indicators of muscular strength, and their decline is almost consistently linked to an elevated risk of total mortality [11, 12, 13, 14, 15, 16]. Leg‐press strength is a parameter that has been less frequently utilized in conventional assessments and quantifies the simultaneous extension of the hip, knee and ankle joints in both lower extremities. Thus, leg‐press strength includes most of the major leg muscles, including the gluteus muscles, as well as the femoral and calf muscles. Accordingly, the volume of muscle mass evaluated in the leg‐press strength is substantially greater than in handgrip or quadriceps strength, and the leg‐press strength may fully capture the functional capacity of the lower extremities required for complex movements [17]. According to the Asian Working Group for Sarcopenia (AWGS) 2025 consensus update, calf circumference is now the key primary assessment tool for sarcopenia case‐finding [18]. Notably, leg‐press strength is the only metric among the conventional muscle strength assessments shown above that incorporates the functional contribution of the calf muscles.

To the best of our knowledge, two cohorts to date have evaluated the association between leg‐press strength and mortality. One cohort demonstrated that higher leg‐press strength in older adults is linked to a lower total mortality risk [14]. Ruiz et al. targeted men only and combined leg‐press strength with upper limb strength, as measured by the bench press, and reported its association with mortality. Their upper limb and leg‐press combined analysis, without women, reported that strong muscle strength was linked to reduced mortality risks of all‐cause and cancer, while cardiovascular disease mortality was not associated [17, 19]. However, both cohorts used estimated maximum leg‐press values calculated from load and repetitions; consequently, very few studies have directly assessed leg‐press strength.

This study analysed a 16‐year follow‐up of the Tsurugaya Project, a community‐based cohort comprising approximately 1100 Japanese adults aged ≥ 70 [16, 20]. Leg‐press strength, defined by directly measured maximal values, was integrated with the GNRI to evaluate its prognostic value. This study aimed to clarify the following issues: (1) Whether directly measured leg‐press strength, rather than conventional estimated values, can predict the total mortality risk in a prospective cohort of older men and women. (2) Whether the combination of muscle strength (MS) by leg‐press and nutritional status (GNRI), termed MS‐GNRI, can more accurately stratify individuals at high risk for all‐cause mortality.

## 2 Methods

### 2.1 Participants

We analysed the data from the Tsurugaya Project, a Japanese regional geriatric cohort study involving individuals over 70 years old, starting from 2002 [21]. The study recruited the general older population residing in the Tsurugaya area of Sendai City, Miyagi Prefecture. The Ethics Committee of Tohoku University Graduate School of Medicine approved the Tsurugaya Project (Approval Number: 2002040). All participants provided informed consent.

### 2.2 Sociodemographic and Medical Information

Sociodemographic information, including age, gender, smoking history, alcohol history and medical history (hypertension, diabetes, cancer, cardiovascular disease, liver disease and kidney disease), was collected at baseline using standardized questionnaires. BMI was evaluated using weight and height. Cognitive function was judged by the Mini‐Mental State Examination (MMSE). The serum albumin levels were quantified by a clinical testing laboratory.

### 2.3 Leg‐Press Strength Measurement

A horizontally aligned leg extension device (Combi Anaeropress3500, Combi Co., Tokyo, Japan) evaluated leg‐press strength (Watts). Participants sat on the device with their feet positioned and secured on the pedal, maintaining a knee angle of 90°. They exerted maximal power on the pedal as rapidly as possible. A total of five measurements of leg‐press strength were conducted, and the highest values were recorded [20]. To adjust for body size, leg‐press strength was divided by BMI (W/kg/m^2^). Participants were then stratified into high and low leg‐press strength groups for each sex. The cutoff values were set at 272.63 (W/kg/m^2^) for males and 129.78 (W/kg/m^2^) for females. To categorize participants as shown above, the optimal cutoff values for leg‐press strength were determined using the maximally selected rank statistics with all‐cause mortality as the time‐to‐event outcome (via the survminer package, Version 0.5.0, in R, Version 4.4.2).

### 2.4 GNRI

Nutritional status in older adults was judged by calculating the GNRI [3, 22]. The formula for GNRI was: (serum albumin [g/L] × 1.489) + ([measured body weight/optimal body weight] × 41.7). We set the ratio of measured body weight/optimal body weight = 1 when the measured body weight was greater than optimal body weight. The optimal body weight was gender‐specific, determined by Lorentz equations, which were formulated as follows for males: height (cm) − 100 – ([height −150]/4), and for females: height (cm) − 100 – ([height −150]/2.5). While conventional GNRI cutoff values were originally established to assess the risk of complications and mortality in hospitalized older patients, this study involved a community‐dwelling population followed over a 16‐year period. To optimize prognostic accuracy for the long‐term observation, the optimal cutoff value for GNRI was determined using the maximally selected rank statistics with a 16‐year all‐cause mortality as the time‐to‐event outcome, using the survminer package, Version 0.5.0, in R, Version 4.4.2. This yielded a study‐specific cutoff value of 105.11, which categorized participants into good and poor GNRI groups.

### 2.5 Construction of the MS–GNRI Joint Indicator

Based on the predefined cutoff values for leg‐press strength and GNRI, a joint indicator, MS‐GNRI, was constructed. The MS‐GNRI stratified participants into four distinct groups based on the combination of baseline leg‐press strength (classified as ‘high’ or ‘low’) and GNRI (classified as ‘good’ or ‘poor’). The optimal cutoff values for leg‐press strength were 272.63 and 129.78 (W/kg/m^2^) for males and females, respectively. The optimal cutoff value for the GNRI was 105.11. Participants were categorized into the following four groups: high leg‐press strength and good GNRI (high‐strength/good‐GNRI), high leg‐press strength and poor GNRI (high‐strength/poor‐GNRI), low leg‐press strength and good GNRI (low‐strength/good‐GNRI) and low leg‐press strength and poor GNRI (low‐strength/poor‐GNRI).

### 2.6 Outcome Follow‐Up

The outcomes were all‐cause mortality. The mortality data were collected from the Sendai Municipal Authority, covering the period until 1 July 2018 [16].

### 2.7 Statistical Analysis

Categorical variables underwent chi‐square testing, and differences in continuous variables were assessed with Student's *t*‐test to examine baseline characteristics stratified by MS‐GNRI. Survival curves were estimated using the Kaplan–Meier method and compared between groups using the log‐rank test. Cox proportional hazards models were employed to assess the association between MS‐GNRI and all‐cause mortality, with results expressed as hazard ratios (HRs) and 95% confidence intervals (CIs), setting the high leg‐press strength and good GNRI group as the reference. To evaluate the association between MS‐GNRI and all‐cause mortality, two Cox proportional hazards models were utilized. Model 1: without adjustments. Model 2: adjusted with sex, age, smoking history, Timed Up and Go Test (TUG), MMSE, past history of hypertension, diabetes, cardiovascular disease, liver disease and kidney disease. Similar evaluations were conducted for groups categorized individually by leg‐press strength and GNRI, using the high‐strength and good GNRI groups as respective references. To preserve the continuous nature of the variables, GNRI and leg‐press strength were analysed as continuous variables using Cox proportional hazards regression models. GNRI was analysed in the overall cohort, whereas leg‐press strength was analysed separately for men and women. The same adjustment strategies as described above were applied, and HRs (95% CIs) were estimated. The discriminative ability was quantified using Harrell's concordance index (C‐index) to validate model performance. Furthermore, time‐dependent area under the curve (AUC) were calculated at 8 and 12 years of follow‐up. Improvements in risk prediction after adding MS–GNRI to the baseline model were further assessed using the net reclassification improvement (NRI). Sensitivity analyses evaluated the robustness of the findings. Participants who did not survive the initial 2‐year follow‐up period were omitted, and the association was reanalysed as sensitivity analyses. Statistical processing was conducted using R Software Version 4.4.2. Survival data were analysed with the survival package (Version 3.8.3); subsequently, Kaplan–Meier survival curves were plotted and refined using the survminer (Version 0.5.0) and ggplot2 (Version 3.5.2) packages. Statistical significance was defined as a two‐tailed *p* value < 0.05.

## 3 Results

**FIGURE 1:**
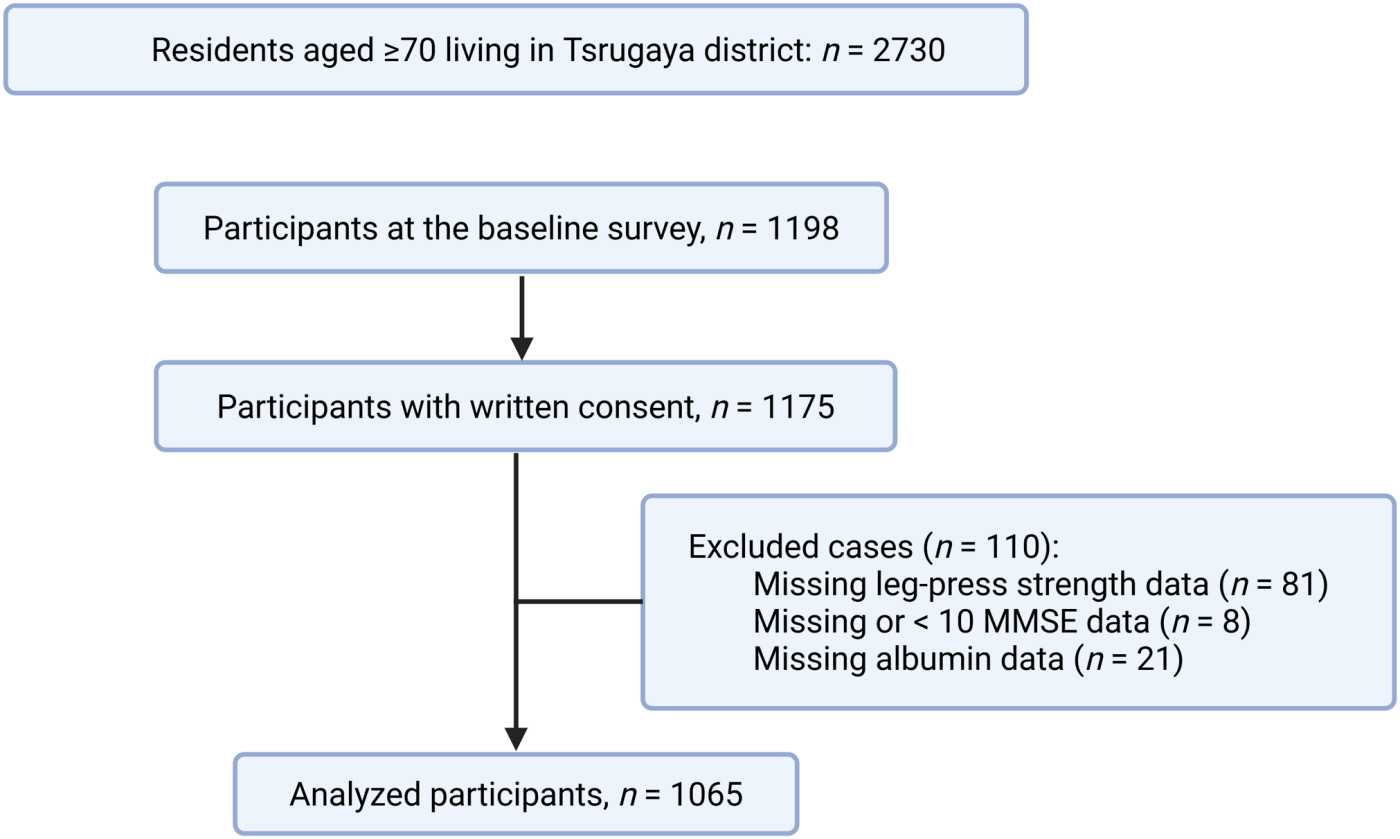
Eligible participants in this research. MMSE, Mini‐Mental State Examination.

In 2002, the Tsurugaya cohort invited 2730 community‐dwelling individuals aged ≥ 70 years in the Tsurugaya area. A total of 1198 participants joined, and 1175 provided informed consent. Individuals without leg‐press strength data (*n* = 81), without or low MMSE data (*n* = 8) or without albumin values (*n* = 21) were removed, and a total of 1065 participants were analysed (Figure 1).

**FIGURE 2:**
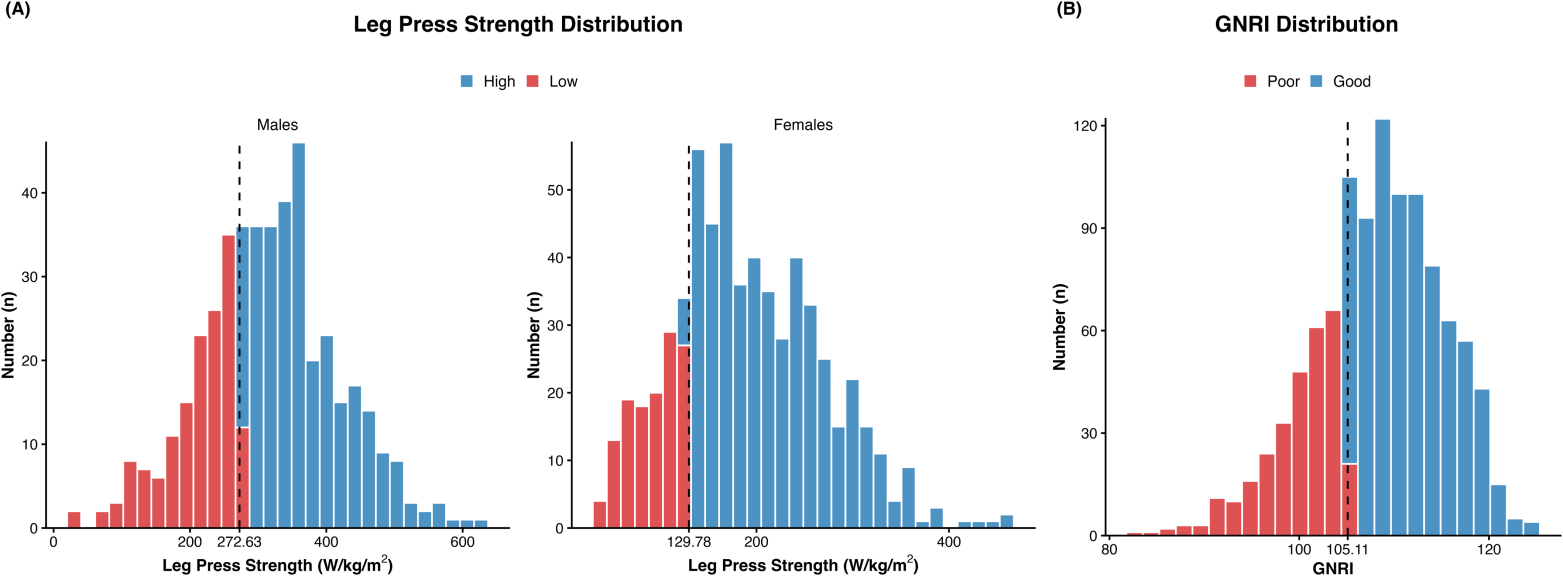
Optimal leg‐press strength and GNRI cutoff values. (A) Gender‐specific distribution of leg‐press strength, with indicated cutoffs determined by the maximally selected rank statistics for males (272.63 W/kg/m^2^, left panel) and females (129.78 W/kg/m^2^, right panel). (B) GNRI distribution and its optimal threshold (105.11) determined by the same statistics. GNRI, Geriatric Nutritional Risk Index.

The separate cutoff values of leg‐press strength for men and women were analysed using the maximally selected rank statistics with all‐cause death as the endpoint. The weaker group, those below the thresholds, accounted for 26.29% of the total (Figure 2A). For nutritional evaluation, we used participants' weight, height and albumin concentrations and calculated GNRI. In contrast to traditional GNRI cutoffs for predicting short‐term outcomes in hospitalized patients, we applied a sex‐neutral threshold of 105.11 for this community‐dwelling cohort. This threshold was optimized using the maximally selected rank statistics with 16‐year all‐cause mortality as the endpoint to maximize its long‐term predictive accuracy, dividing participants into good and poor groups (Figure 2B). The poor group included 29.95% of the total participants. Prior to combining the muscle strength and nutritional indices, we examined the correlation between leg‐press strength and GNRI. The correlation coefficient between leg‐press strength and GNRI was 0.096, indicating a negligible correlation. Furthermore, the variance inflation factor was 1.009, indicating no multicollinearity issues among these variables. Accordingly, we integrated muscle strength (MS) and GNRI into a single index, which we designated as MS‐GNRI. To develop the MS‐GNRI, we first categorized participants into two groups for each index: high and low for leg‐press strength and good and poor for GNRI. Subsequently, these classifications were combined as MS‐GNRI to stratify all participants into the following four groups: (1) high leg‐press strength and good GNRI (high‐strength/good‐GNRI, *n* = 589), (2) high leg‐press strength and poor GNRI (high‐strength/poor‐GNRI, *n* = 196), (3) low leg‐press strength and good GNRI (low‐strength/good‐GNRI, *n* = 157) and (4) low leg‐press strength and poor GNRI (low‐strength/poor‐GNRI, *n* = 123). Table 1 demonstrates the participants' initial characteristics divided into four groups. The proportion of male participants was lowest in the high‐strength/good‐GNRI group and increased progressively across the high‐strength/poor‐GNRI, low‐strength/good‐GNRI and low‐strength/poor‐GNRI groups. A similar increasing trend was observed for age and the prevalence of current smokers across these four groups. Significant differences were observed in MMSE scores among the groups; however, the mean scores in all groups remained above 26, exceeding the clinical cutoff for dementia (23 points). Regarding medical history, significant differences were identified in the prevalence of hypertension, diabetes mellitus and cardiovascular disease. During the 16‐year follow‐up period, a total of 415 all‐cause deaths were recorded (Table 1).

**TABLE 1:** Groups are stratified by leg press strength and Geriatric Nutritional Risk Index (GNRI) joint indicator (MS‐GNRI): high leg press strength and good GNRI (high + good), high leg press strength and poor GNRI (high + poor), low leg press strength and good GNRI (low + good) as well as low leg press strength and poor GNRI (low + poor). Values are reported as mean ± standard deviation (SD) or *n* (%).

		MS‐GNRI group				
Variable	Overall *N* = 1065	High + good *n* = 589 (55.3%)	High + poor *n* = 196 (18.4%)	Low + good *n* = 157 (14.7%)	Low + poor *n* = 123 (11.5%)	*p*
**Male, *n* (%)**	448 (42.1)	221 (37.5)	77 (39.3)	74 (47.1)	76 (61.8)	< 0.001
**Age (years)**	75.67 (4.81)	74.62 (4.08)	75.43 (4.63)	77.34 (5.10)	78.98 (5.82)	< 0.001
**Smoking history, *n* (%)**	460 (43.2)	235 (39.9)	79 (40.3)	77 (49.0)	69 (56.1)	0.003
**Alcohol history, *n* (%)**	428 (40.2)	253 (43.0)	69 (35.2)	59 (37.6)	47 (38.2)	0.208
**BMI (kg/m** ^**2**^ **)**	23.88 (3.36)	25.25 (2.68)	21.06 (1.95)	25.35 (3.12)	19.94 (2.28)	< 0.001
**MMSE**	27.25 (2.83)	27.57 (2.55)	27.59 (2.22)	26.12 (3.95)	26.59 (2.78)	< 0.001
**Albumin (g/dL)**	4.33 (0.27)	4.42 (0.23)	4.15 (0.22)	4.39 (0.27)	4.13 (0.26)	< 0.001
**Timed Up and Go Test (s)**	9.66 (2.85)	9.11 (1.70)	8.99 (1.78)	11.49 (4.39)	11.12 (4.37)	< 0.001
**Medical history**						
Hypertension, *n* (%)	398 (37.4)	241 (40.9)	59 (30.1)	64 (40.8)	34 (27.6)	0.004
Diabetes, *n* (%)	146 (13.7)	75 (12.7)	18 (9.2)	33 (21.0)	20 (16.3)	0.009
Cancer, *n* (%)	72 (6.8)	39 (6.6)	11 (5.6)	14 (8.9)	8 (6.5)	0.660
CVD, *n* (%)	162 (15.2)	80 (13.6)	22 (11.2)	34 (21.7)	26 (21.1)	0.007
Liver disease, *n* (%)	77 (7.2)	42 (7.1)	16 (8.2)	9 (5.7)	10 (8.1)	0.817
Kidney disease, *n* (%)	72 (6.8)	46 (7.8)	12 (6.1)	8 (5.1)	6 (4.9)	0.469

Abbreviations: CVD, cardiovascular disease; MMSE, Mini‐Mental State Examination.

**FIGURE 3:**
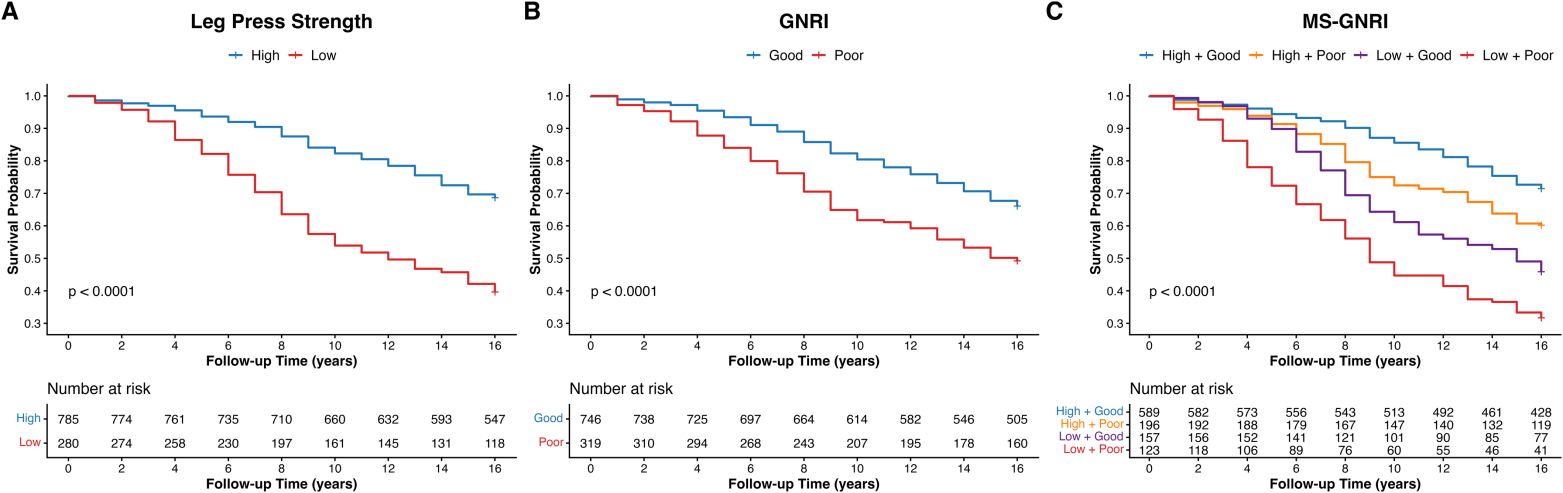
Kaplan–Meier curves illustrating survival probabilities for overall mortality: (A) Leg‐press strength: high versus low, cutoffs for males (272.63 W/kg/m^2^) and females (129.78 W/kg/m^2^). (B) GNRI: good versus poor (threshold: 105.11). (C) Groups are defined as follows: high + good (high leg press strength and good GNRI), high + poor (high leg press strength and poor GNRI), low + good (low leg press strength and good GNRI), and low + poor (low leg press strength and poor GNRI). MS‐GNRI: a composite index based on muscle strength (MS; leg‐press strength) and the Geriatric Nutritional Risk Index (GNRI).

Figure 3 illustrated the Kaplan–Meier survival estimates for the study population stratified into two groups based on leg‐press strength (A) and GNRI (B) and into four groups based on the MS‐GNRI (C). Regarding leg‐press strength, the high‐strength group demonstrated significantly better survival rates than the low‐strength group (Figure 3A). The 1‐year mortality rates (95% CI) were 1.40% (0.58–2.22) in the high group and 2.14% (0.43–3.82) in the low group. The 5‐year mortality rates were 6.37% (4.65–8.06) and 17.86% (13.25–22.22), respectively. In terms of nutritional status, the good‐GNRI group had a higher survival probability than the poor‐GNRI group (Figure 3B). The 1‐year mortality rates (95% CI) were 1.07% (0.33–1.81) in the good group and 2.82% (0.99–4.62) in the poor group. The 5‐year mortality rates were 6.57% (4.77–8.33) and 15.99% (11.87–19.91), respectively. The Kaplan–Meier analysis for the MS‐GNRI revealed a clear gradient in survival probabilities across the four categories. The best survival outcome was observed in the high‐strength/good‐GNRI group, while the worst outcome was found in the low‐strength/poor‐GNRI group (Figure 3C). The 1‐year mortality rates (95% CI) were 1.19% (0.31–2.06) in the high‐strength/good‐GNRI group, 2.04% (0.04–4.00) in the high‐strength/poor‐GNRI group, 0.64% (0.00–1.87) in the low‐strength/good‐GNRI group and 4.07% (0.51–7.49) in the low‐strength/poor‐GNRI group. The 5‐year mortality rates were 5.60% (3.73–7.44) in the high‐strength/good‐GNRI group, 8.67% (4.65–12.53) in the high‐strength/poor‐GNRI group, 10.19% (5.33–14.80) in the low‐strength/good‐GNRI group and 27.64% (19.29–35.13) in the low‐strength/poor‐GNRI group. Interestingly, the high‐strength/poor‐GNRI group demonstrated a trend of better survival rate compared to the low‐strength/good‐GNRI group (*p* < 0.01). Table 2 showed the HRs for all‐cause mortality across the four MS‐GNRI groups, with the high‐strength/good‐GNRI group as reference. In the nonadjusted model (Model 1), the HRs (95% CIs) for all‐cause mortality were 1.55 (1.19–2.03) for the high‐strength/poor‐GNRI group, 2.37 (1.83–3.08) for the low‐strength/good‐GNRI group and 3.78 (2.91–4.92) for the low‐strength/poor‐GNRI group. A stepwise increase in risk was observed across these four groups (*p* for trend < 0.001), with a significant difference between the high‐strength/poor‐GNRI and the low‐strength/good‐GNRI groups (*p* < 0.01). Adjustments were made for sex, age, smoking history, TUG, MMSE and medical history (hypertension, diabetes, cardiovascular disease, liver disease and kidney disease) from Table 1 (Model 2). The HRs (95% CIs) were 1.55 (1.18–2.03) for the high‐strength/poor‐GNRI group, 1.69 (1.29–2.23) for the low‐strength/good‐GNRI group and 2.47 (1.78–3.13) for the low‐strength/poor‐GNRI group, with a stepwise increase in risk across these categories (*p* for trend < 0.001). Although the stepwise increase persisted, the difference between the high‐strength/poor‐GNRI and low‐strength/good‐GNRI groups became nonsignificant after adjustment. Furthermore, participants were stratified by leg‐press strength and GNRI, respectively. Compared with their corresponding reference groups (high‐strength and good‐GNRI), adjusted HRs were 1.81 (1.45–2.25) for the low‐strength group (Table 3A) and 1.59 (1.30–1.95) for the poor‐GNRI group (Table 3B; both *p* < 0.01).

**TABLE 2:** Relationship between MS‐GNRI groups and all‐cause mortality. Groups are defined as follows: high + good (high leg press strength and good GNRI), high + poor (high leg press strength and poor GNRI), low + good (low leg press strength and good GNRI) and low + poor (low leg press strength and poor GNRI). Data are presented as hazard ratios (95% confidence intervals). Model 1: defined as a model without adjustment, Model 2: adjusted for gender, age, smoking history, Mini‐Mental State Examination (MMSE), Timed Up and Go Test, past history of hypertension, diabetes, cardiovascular disease, liver disease and kidney disease. MS‐GNRI: a composite index based on muscle strength (MS; leg press strength) and the Geriatric Nutritional Risk Index (GNRI).

	High + good	High + poor	Low + good	Low + poor	*p* for trend
**All‐cause deaths (*n*)**	**168**	**78**	**85**	**84**	
Model 1	1.00 (reference)	1.55 (1.19–2.03) *	2.37 (1.83–3.08)**	3.78 (2.91–4.92)**	< 0.001
Model 2	1.00 (reference)	1.55 (1.18–2.03) *	1.69 (1.29–2.23)**	2.47 (1.78–3.13)**	< 0.001

* *p* < 0.01.

** *p* < 0.001 versus high leg press strength and good GNRI group.

**TABLE 3:** Relationship of (A) leg‐press strength and (B) GNRI with all‐cause mortality. Participants were stratified into two groups based on leg‐press strength (high/low) and GNRI (good/poor). Data are presented as hazard ratios (95% confidence intervals). Model 1: defined as a model without adjustment, Model 2: adjusted for gender, age, smoking history, Mini‐Mental State Examination (MMSE), Timed Up and Go Test, past history of hypertension, diabetes, cardiovascular disease, liver disease and kidney disease.

(A)			
	High	Low	*p* value
**Number (*n*)**	**799**	**266**	
**All‐cause deaths (*n*)**	**259**	**156**	
Model 1	1.00 (reference)	2.38 (1.95–2.90)	< 0.001
Model 2	1.00 (reference)	1.81 (1.45–2.25)	< 0.001

(B)			
	Good	Poor	*p* value
**Number (*n*)**	**746**	**319**	
**All‐cause deaths (*n*)**	**253**	**162**	
Model 1	1.00 (reference)	1.80 (1.47–2.19)	< 0.001
Model 2	1.00 (reference)	1.59 (1.30–1.95)	< 0.001

To evaluate whether leg‐press strength can predict the 16‐year total mortality risk when treated as a continuous variable, we estimated the HRs. Since leg‐press strength differs between sexes, the analyses were conducted separately for males and females. Model 1 was a crude model, and Model 2 was adjusted for sex, age, smoking history, TUG, MMSE and medical history (hypertension, diabetes, cardiovascular, liver and kidney diseases). In Model 2, a one‐unit increase in leg‐press strength was significantly associated with a decreased risk of mortality, with HRs (95% CIs) of 0.9965 (0.9949–0.9981) for males and 0.9975 (0.9951–0.9998) for females (Table S1). Similarly, when examining GNRI as a continuous variable, a one‐unit increase was significantly associated with a reduced risk of mortality, with the HR (95% CI) of 0.9726 (0.9593–0.9860) (Table 1).

Considering the potential for reverse causality—specifically that pre‐existing illness at baseline may have impaired both strength and nutritional indicators—we conducted a sensitivity analysis excluding 30 participants who died during the first 2 years of the study. The Kaplan–Meier survival curves showed a similar trend to the results that included the first 2 years of follow‐up (Figure S1). The high‐strength/good‐GNRI group (*n* = 577) exhibited the highest survival rate, followed by a stepwise decline in survival for the following groups: high‐strength/poor‐GNRI (*n* = 190), low‐strength/good‐GNRI (*n* = 154) and low‐strength/poor‐GNRI (*n* = 114). With the high‐strength/good‐GNRI group as the reference, the adjusted HRs (95% CIs) were 1.55 (1.17–2.05) for high‐strength/poor‐GNRI, 1.80 (1.36–2.38) for low‐strength/good‐GNRI and 2.39 (1.78–3.21) for low‐strength/poor‐GNRI (Table S2). A stepwise increase in mortality risk was identified across these categories (*p* for trend < 0.001).

We further assessed whether the combination of muscle strength and GNRI improved the accuracy for mortality risk stratification compared with each indicator alone using the concordance index (C‐index). A C‐index of 0.5 indicates no discriminative power, while a score of 1.0 represents perfect discrimination. Thus, the effectiveness of the model in risk stratification is represented by higher C‐index values. Furthermore, the Net Reclassification Improvement (NRI) was calculated to quantify the degree of improvement in risk stratification. The NRI quantifies the extent to which individuals are correctly reclassified into higher risk categories by the updated model compared with the reference model. The C‐index was 0.54 for muscle strength and 0.59 for GNRI. The MS‐GNRI improved the index to 0.63, suggesting that the combination significantly enhanced predictive accuracy compared with either indicator alone (*p* < 0.01). Significant improvements in risk reclassification were observed for the MS‐GNRI, with NRI values (95% CI) of 0.62 (0.47–0.75) over muscle strength and 0.41 (0.17–0.62) over GNRI (*p* < 0.01). Finally, we examined the time‐dependent area under the curve (AUC) at 8 and 12 years of follow‐up. At 8 years, the AUC was 0.65 for muscle strength, 0.61 for GNRI and 0.70 for MS‐GNRI; the MS‐GNRI significantly improved predictive accuracy compared with either indicator alone (*p* < 0.01). Similar trends were observed at 12 years, with MS‐GNRI showing better predictive accuracy than each individual indicator (*p* < 0.01), with AUC of 0.64 for muscle strength, 0.59 for GNRI and 0.67 for MS‐GNRI.

## 4 Discussion

By analysing a cohort of community‐dwelling older adults, the current research demonstrated a significant link between directly measured leg‐press strength and all‐cause mortality—a relationship that was previously largely unexplored. Furthermore, we integrated muscle strength with nutritional status, a combination rarely investigated in the general elderly population for the long term. Our findings revealed that individuals with both high muscle strength and good nutritional status exhibited the highest survival rates, whereas those with low muscle strength and poor nutrition showed the worst survival outcomes. After adjustment, the high‐muscle‐strength and poor‐nutritional group exhibited a mortality risk comparable to that of the low‐muscle‐strength and good‐nutritional group. Our findings from the C‐index and NRI suggest that the combination of muscle strength and GNRI provides superior predictive accuracy for mortality risk stratification compared with either indicator used independently.

The analysis by Ruiz et al. using leg‐press strength with upper limb strength calculated the muscle strength and consisted of only men [17, 19]. Our measurements differed in that we directly measured the muscle strength, did not include upper limb strength and included both men and women. However, similarly, in outcomes, we found elevated risks of all‐cause mortality. The low leg‐press strength group showed worse all‐cause mortality risk in both men and women in this study, consistent with the previous analysis that estimated the leg‐press strength [14]. While the AWGS 2025 update emphasizes calf circumference for sarcopenia case‐finding, data on calf‐muscle strength and mortality are limited. The present study on leg‐press strength, incorporating the calf muscles, may bridge this gap by providing supportive evidence.

As hypothesized, the four groups stratified by leg‐press strength and nutritional status showed distinct survival gradients: The high‐strength/good‐GNRI group exhibited the best survival, while the low‐strength/poor‐GNRI group showed the worst. A higher survival probability was initially observed in the high‐strength/poor‐GNRI group than in the low‐strength/good‐GNRI group; however, this association became nonsignificant after adjustment. Among the baseline variables presented in Table 1, those with a *p* value < 0.1 were selected as covariates for the Cox proportional hazards models, such as MMSE. Additionally, liver disease and kidney disease were included as potential confounding factors, given their known physiological impact on serum albumin levels. The current findings suggest that while adequate nutrition is essential for longevity, preserved muscle strength may contribute to extending life expectancy.

While muscle strength and GNRI provided a baseline for risk assessment, their integration into the MS‐GNRI enhanced the C‐index from the 0.5 range to the 0.6 range. This statistically significant improvement suggests that the combined index might complement traditional nutritional assessment for stratifying mortality risk. Compared with muscle strength, MS‐GNRI yielded an NRI of 0.62, suggesting that predictive performance could be enhanced by 62%. Furthermore, compared to GNRI alone, the value was 0.41, suggesting a 41% improvement in accuracy. The improvement in the NRI demonstrated by MS‐GNRI over each individual component suggests the potential importance of this combination in mortality risk stratification among the elderly.

In East Asian populations, including Japan, the three primary causes of mortality include malignancy, cardiovascular disease and pneumonia [23]. All‐cause mortality was defined as the primary outcome in this study to assess its association with low leg‐press strength. While cause‐specific mortality was not individually analysed, the potential implications for specific causes of death were discussed according to previous findings. A previous cohort study indicated that diminished leg‐press strength exacerbates the risk of cancer death, yet it appeared independent of cardiovascular disease mortality. The relationship with pneumonia mortality, however, remains poorly understood. When focusing on quadriceps strength—a component of the leg press—only limited research has identified a potential association with pneumonia mortality [24]. One study that analysed the very old population aged 80 and above reported that quadriceps weakness increased the risk of pneumonia mortality with a 4‐year follow‐up [25]. In contrast, malnutrition exacerbates the mortality risks over all major causes, such as cancer, cardiovascular disease and pneumonia [21, 26]. These findings suggest that low leg‐press strength, either independently or in combination with other factors, may elevate the risk of mortality from cancer and pneumonia—two of the three leading causes of death. While the independent association between weak leg‐press strength and cardiovascular disease mortality remains unclear, our findings suggest that its combination with malnutrition may elevate the risk of cardiovascular disease‐related death.

### 4.1 Study Limitations

Although a correlation between leg‐press strength and all‐cause mortality was observed, it was unable to determine whether leg‐press strength serves as a superior predictor of mortality compared to other measures of muscular strength, such as handgrip or quadriceps strength, without a head‐to‐head comparison. Furthermore, as this research analysed a Japanese cohort, the findings primarily reflect the link between muscle strength and nutritional status in East Asian individuals. Thus, the applicability of the above data to other ethnic populations remains to be established. Although study‐specific cutoffs were utilized to maximize prognostic accuracy for this 16‐year follow‐up, further validation in independent cohorts is warranted to confirm the generalizability of these thresholds.

## 5 Conclusions

Few studies have linked directly measured maximum leg‐press strength with all‐cause mortality. Furthermore, there has been a paucity of long‐term prospective cohort studies examining the integrated impact of muscle strength and nutritional status on all‐cause mortality in general older individuals. When utilizing leg‐press strength as a measure of the overall strength of most of the major muscles of the right and left lower extremities, its weakness was related to elevated all‐cause mortality risk. Survival probability was highest in the high‐strength/good‐GNRI group and lowest in the low‐strength/poor‐GNRI group. The high‐strength/poor‐GNRI and low‐strength/good‐GNRI groups exhibited comparable, intermediate survival rates. These findings imply that preserved muscle strength is a critical determinant in promoting longevity, potentially playing a substantial role alongside nutritional maintenance. Furthermore, the MS‐GNRI demonstrated enhanced accuracy for mortality risk stratification compared to using either metric independently, highlighting the clinical utility of assessing both indices in high‐risk older adults. Future challenges include whether increasing leg‐press strength can reduce the all‐cause mortality risks.

## Funding

This research received financial support from a Grant‐in‐Aid for Scientific Research provided by the JSPS to T.O. (18K08133 and 22H029643) and S E. (24K02778 and 22K19760) and JST SICORP to S.E. (JPMJSC2308). The General Insurance Association of Japan to T.O., Research Funding for Longevity Science from the National Center for Geriatrics and Gerontology (25‐29) and the Grant from the Japan Association of Geriatric Health Service Facilities (Dysphagia Statement Project to S.E.).

## Ethics Statement

The Ethics Committee of Tohoku University Graduate School of Medicine approved the Tsurugaya Project (Approval Number: 2002040). All participants provided informed consent. All the procedures were conducted under the Declaration of Helsinki.

## Conflicts of Interest

The authors declare no conflicts of interest.

## Supporting information


**Figure S1:** Cumulative survival rates according to MS‐GNRI groups. Analyses exclude participants who died within the initial two‐year follow‐up period (*n* = 30). MS‐GNRI: A composite index based on muscle strength (MS; leg‐press strength) and the Geriatric Nutritional Risk Index (GNRI).

**Table S1:** Association of continuous sex‐specific leg‐press strength and GNRI with all‐cause mortality. Hazard ratios (HRs) are presented per 1‐unit increase in leg‐press strength and GNRI, with corresponding 95% confidence intervals (CIs). Model 1: defined as a model without adjustment. Model 2: adjusted for gender, age, smoking history, Mini‐Mental State Examination (MMSE), Timed Up and Go Test and past history of hypertension, diabetes, cardiovascular disease, liver disease and kidney disease.

**Table S2:** Relationship between MS‐GNRI groups and all‐cause mortality, excluding participants who died during the first 2 years of the study (*n* = 30). Groups are defined as follows: high + good (high leg press strength and good GNRI), high + poor (high leg press strength and poor GNRI), low + good (low leg press strength and good GNRI) and low + poor (low leg press strength and poor GNRI). Data are presented as hazard ratios (95% confidence intervals). Model 1: defined as a model without adjustment, Model 2: adjusted for gender, age, smoking history, Mini‐Mental State Examination (MMSE), Timed Up and Go Test, past history of hypertension, diabetes, cardiovascular disease, liver disease and kidney disease. *p < 0.01, ^§^ *p* < 0.001 versus high leg press strength and good GNRI group. MS‐GNRI: A composite index based on muscle strength (MS; leg press strength) and the Geriatric Nutritional Risk Index (GNRI).

## Data Availability

The data that support the findings of this study are available on request from the corresponding author. The data are not publicly available due to privacy or ethical restrictions.
