# Supplementary material for: Integrating Leg‐Press Strength and Nutritional Status Improves 16‐Year Mortality Risk Stratification in General Older Adults
Source: J Cachexia Sarcopenia Muscle. 2026 Sep 16;17(5):e70375. doi: 10.1002/jcsm.70375 (PMC13580464; doi:10.1002/jcsm.70375)
Supplement: Supplementary file 1 [file JCSM-17-e70375-s001.docx]

|  | **High + Good** | **High + Poor** | **Low + Good** | **Low + Poor** | ***p* for trend** |
| --- | --- | --- | --- | --- | --- |
| **All-cause deaths (n)** | **161** | **74** | **84** | **79** |  |
| Model 1 | 1.00 (Reference) | 1.55 (1.17-2.05) * | 2.51 (1.92-3.28) ^§^ | 3.77 (2.86-4.97) ^§^ | < 0.001 |
| Model 2 | 1.00 (Reference) | 1.55 (1.17-2.05) * | 1.80 (1.36-2.38) ^§^ | 2.39 (1.78-3.21) ^§^ | < 0.001 |

Supplemental Table 2: Relationship between MS-GNRI groups and all-cause mortality, excluding participants who died during the first two years of the study (n = 30). Groups are defined as follows: High + Good (high leg press strength and good GNRI), High + Poor (high leg press strength and poor GNRI), Low + Good (low leg press strength and good GNRI), and Low + Poor (low leg press strength and poor GNRI). Data are presented as hazard ratios (95% confidence intervals). Model 1: defined as a model without adjustment, Model 2: adjusted for gender, age, smoking history, Mini-Mental State Examination (MMSE), Timed Up and Go Test, past history of hypertension, diabetes, cardiovascular disease, liver disease and kidney disease. * *p* < 0.01, ^§^ *p* < 0.001 vs. high leg press strength and good GNRI group. MS-GNRI: A composite index based on muscle strength (MS; leg press strength) and the Geriatric Nutritional Risk Index (GNRI).
