# Supplementary material for: Integrating Leg‐Press Strength and Nutritional Status Improves 16‐Year Mortality Risk Stratification in General Older Adults
Source: J Cachexia Sarcopenia Muscle. 2026 Sep 16;17(5):e70375. doi: 10.1002/jcsm.70375 (PMC13580464; doi:10.1002/jcsm.70375)
Supplement: Supplementary file 2 [file JCSM-17-e70375-s003.docx]

|  | **HR (95% CI)** | ***p* value** |
| --- | --- | --- |
| **Leg Press Strength - Male** |  |  |
| Model 1 | 0.9947 (0.9933-0.9961) | < 0.001 |
| Model 2 | 0.9965 (0.9949-0.9981) | < 0.001 |
| **Leg Press Strength - Female** |  |  |
| Model 1 | 0.9949 (0.9928-0.9969) | < 0.001 |
| Model 2 | 0.9975 (0.9951-0.9998) | 0.032 |
| **GNRI** |  |  |
| Model 1 | 0.9591 (0.9468-0.9716) | < 0.001 |
| Model 2 | 0.9726 (0.9593-0.9860) | < 0.001 |

Supplemental Table 1: Association of continuous sex-specific leg-press strength and GNRI with all-cause mortality. Hazard ratios (HRs) are presented per 1-unit increase in leg-press strength and GNRI, with corresponding 95% confidence intervals (CIs). Model 1: defined as a model without adjustment. Model 2: adjusted for gender, age, smoking history, Mini-Mental State Examination (MMSE), Timed Up and Go Test, and past history of hypertension, diabetes, cardiovascular disease, liver disease, and kidney disease.
